# Determining *Mycobacterium tuberculosis* Infection among BCG-Immunised Ugandan Children by T-SPOT.TB and Tuberculin Skin Testing

**DOI:** 10.1371/journal.pone.0047340

**Published:** 2012-10-15

**Authors:** Gyaviira Nkurunungi, Jimreeves E. Lutangira, Swaib A. Lule, Hellen Akurut, Robert Kizindo, Joseph R. Fitchett, Dennison Kizito, Ismail Sebina, Lawrence Muhangi, Emily L. Webb, Stephen Cose, Alison M. Elliott

**Affiliations:** 1 Co-infection Studies Programme, Medical Research Council/Uganda Virus Research Institute Uganda Research Unit on AIDS, Entebbe, Uganda; 2 Department of Clinical Research, London School of Hygiene and Tropical Medicine, London, United Kingdom; 3 Department of Infectious Disease Epidemiology, London School of Hygiene and Tropical Medicine, London, United Kingdom; McGill University, Canada

## Abstract

**Background:**

Children with latent tuberculosis infection (LTBI) represent a huge reservoir for future disease. We wished to determine *Mycobacterium tuberculosis (M.tb)* infection prevalence among BCG-immunised five-year-old children in Entebbe, Uganda, but there are limited data on the performance of immunoassays for diagnosis of tuberculosis infection in children in endemic settings. We therefore evaluated agreement between a commercial interferon gamma release assay (T-SPOT.TB) and the tuberculin skin test (TST; 2 units RT-23 tuberculin; positive defined as diameter ≥10 mm), along with the reproducibility of T-SPOT.TB on short-term follow-up, in this population.

**Methodology/Principal Findings:**

We recruited 907 children of which 56 were household contacts of TB patients. They were tested with T-SPOT.TB at age five years and then re-examined with T-SPOT.TB (n = 405) and TST (n = 319) approximately three weeks later. The principal outcome measures were T-SPOT.TB and TST positivity. At five years, 88 (9.7%) children tested positive by T-SPOT.TB. More than half of those that were T-SPOT.TB positive at five years were negative at follow-up, whereas 96% of baseline negatives were consistently negative. We observed somewhat better agreement between initial and follow-up T-SPOT.TB results among household TB contacts (κ = 0.77) than among non-contacts (κ = 0.39). Agreement between T-SPOT.TB and TST was weak (κ = 0.28 and κ = 0.40 for T-SPOT.TB at 5 years and follow-up, respectively). Of 28 children who were positive on both T-SPOT.TB tests, 14 (50%) had a negative TST. Analysis of spot counts showed high levels of instability in responses between baseline and follow-up, indicating variability in circulating numbers of T cells specific for certain *M.tb* antigens.

**Conclusions/Significance:**

We found that T-SPOT.TB positives are unstable over a three-week follow-up interval, and that TST compares poorly with T-SPOT.TB, making the categorisation of children as TB-infected or TB-uninfected difficult. Existing tools for the diagnosis of TB infection are unsatisfactory in determining infection among children in this setting.

## Introduction

Worldwide, tuberculosis (TB) remains one of the most important infectious causes of mortality. In 2010, there were an estimated 8.8 million incident cases and approximately 1.4 million people died from this disease [1]. Uganda is designated by the World Health Organisation (WHO) to be one of the 22 high burden countries for TB. A vast pool of individuals with latent tuberculosis infection (LTBI) persists in developing countries, posing a major barrier to global TB control [2]. The overall lifetime risk of LTBI reactivation is approximately 5–10% among older children and adults, but in infants and young children, the risk of progression to active disease is increased; most disease cases occur within 12 months of infection [3], [4]. Moreover, infection in childhood establishes the reservoir for future epidemics, making proper diagnosis and treatment of LTBI in this vulnerable group important for TB control [5].

For many years, the standard technique used to diagnose LTBI has been the tuberculin skin test (TST). Although the TST has proven to be useful in clinical practice, it has several known limitations [3], [6]. Perhaps the most significant of these is the cross reactivity of the purified protein derivative of tuberculin used in the TST with antigens from several nontuberculous mycobacteria, and also with those from the *Mycobacterium bovis* bacille Calmette-Guérin (BCG) vaccine [7], [8]. This means that the skin test may not reliably discern LTBI from prior immunisation or infection with other mycobacteria. Work done in the last decade has suggested that *in vitro* T-cell based interferon gamma release assays (IGRAs) may offer a suitable alternative approach to LTBI diagnosis [6], [9]. One such assay is an Enzyme-linked immunosorbent spot (ELISpot) assay, commercially known as T-SPOT.TB, whose antigens (early secretory antigenic target 6, ESAT-6 and culture filtrate protein 10, CFP-10) are coded by the Region of Difference 1 (RD1) genes of the *Mycobacterium tuberculosis* (*M.tb*) complex which is absent from the majority of nontuberculous isolates, as well as from BCG [7], [10].

These immunoassays have been widely studied and reviewed in adults and in contacts of infectious cases [3], [9]–[17], yet very little data exist about their performance in children from the general population, and whether their diagnostic accuracy is superior to that of TST, especially in endemic settings. In a recent systematic review and meta-analysis, it was observed that IGRAs are less sensitive and less specific in areas of high TB burden [18]. It is not known whether IGRAs can replace TST surveys as a tool for estimating the annual incidence of infection with *M.tb*. Lack of a “gold standard” reference test for LTBI has made it difficult to assess performance of IGRAs, prompting adoption of surrogate indicators such as evaluation of efficacy of preventive therapy based on test results, predictive value of a test for active TB, correlation with exposure gradient, sensitivity and/or specificity among patients with active TB, and concordance between the IGRA and other LTBI tests [19]. Some studies, mostly in adults, have shown that after *M.tb* exposure, IGRA results fluctuate when serial testing is done [14], [20], [21], and this has been attributed to variations in laboratory procedures, within subject variability and biological and environmental causes [22].

Within the structure of an existing study [23], we set out to measure the prevalence of LTBI among BCG-immunised five-year olds in Entebbe, Uganda, using the T-SPOT.TB assay. We performed repeat T-SPOT.TB assays approximately three weeks later to determine stability of responses, as well as the more conventional TST to allow comparison with T-SPOT.TB results. We present findings detailing T-SPOT.TB and TST test agreement, stability of T-SPOT.TB responses on short-term follow-up, and analysis of spot forming units in *M.tb* exposed and unexposed children in a high prevalence African setting.

## Methods

### Study Design and Participants

This was an observational, cross sectional study. From March 2009 to April 2011, participants were prospectively recruited at the fifth annual visit within the framework of the Entebbe Mother and Baby Study (EMaBS), a population-based birth cohort in Entebbe, Wakiso district, Central Uganda [23]. EMaBS was originally established to evaluate the impact of maternal and childhood helminth infections and of anthelminthic treatment on immune responses to vaccines and childhood infections. The study setting was an area with a moderately high background rate of TB: unpublished data from the Uganda National TB and Leprosy Programme (NTLP) indicates that 929 new pulmonary and extra-pulmonary TB cases were detected in Wakiso district (estimated population 1,205,000) in 2009, an incidence of 77 per 100,000.

Participants in EMaBS were enrolled at the Entebbe Hospital antenatal clinic. Follow-up and enrolment into this sub-study of TB infection was done at the EMaBS outpatient clinic within the hospital grounds. Assessment of the household TB contacts, and verification of the TB cases they were exposed to, was done at the study clinic through verbal interviews of the children’s parents or guardians. T-SPOT.TB assays were performed in the main immunology research laboratory at the Uganda Virus Research Institute, a five minute drive from the hospital. Demographic, socioeconomic, and health-related information was collected prospectively from enrolment through to follow-up at five years. Participants were medically examined and anthropometric measurements recorded. All the children were enrolled into the parent study and into the T-SPOT.TB sub-study reported here following informed and documented written consent by their parents or formal guardian. Children were included in this sub-study if they were five years of age, in good health and BCG-immunised. They were excluded if they had moved outside the study area and could not comply with the required procedures, or if the parent or guardian did not give permission for the additional procedures required for this study. Children enrolled in the larger EMaBS study received BCG as neonates [24], [25]: 94% of these immunisations were given under observation at Entebbe Hospital, the remainder were given elsewhere. Records were present at the EMaBS study clinic. In the event of active disease or suspected TB infection due to positive immunodiagnostic testing, they were referred to a physician for medical examination, chest X-ray, and treatment if required.

Our initial objective for the study was to estimate the prevalence of LTBI in five year olds. At first we anticipated that T-SPOT.TB could be used to accurately achieve this. For confirmation of the results, and of the repeatability of the T-SPOT.TB test, the initial protocol (Protocol 1) identified three groups of children to undergo, in addition to T-SPOT.TB at age five years, a TST and repeat T-SPOT.TB at follow-up three weeks later: 1) children who were T-SPOT.TB-positive at 5 years, 2) children who were reported to have had household contact with a TB patient, no matter the initial test result, and 3) a comparison sample of approximately 50 T-SPOT.TB-negative children, selected as the first T-SPOT.TB-negative child, willing to undergo the additional procedures, who was seen each week by the study doctor (SAL). Once variations between the initial and repeat T-SPOT.TB results from the same individuals became evident, it was clear that we could not rely on the initial positive result to determine the infection status of a child. The protocol was then amended and all children who underwent the T-SPOT.TB test at five years were asked to undergo the follow-up T-SPOT.TB and TST tests (Protocol 2). By doing this, we hoped to determine whether children with current LTBI could be identified by using T-SPOT.TB as a screening assay, followed by confirmation with repeat T-SPOT.TB combined with TST.

The study was approved by ethics committees of the Uganda Virus Research Institute and London School of Hygiene and Tropical Medicine, and by the Uganda National Council for Science and Technology.

### Procedures

#### T-SPOT.TB assays

Blood samples were processed within eight hours of collection. The assays were carried out according to the manufacturer’s instructions (Oxford Immunotec, Abingdon, UK). Briefly, peripheral blood mononuclear cells were isolated by centrifugation, washed twice and the cell concentration was adjusted such that each of four wells of the assay plate contained 250,000 cells. The cells were stimulated for 16–20 hours (under 5% carbon dioxide at 37°C) with media (negative control), phytohaemagglutinin (positive control) or peptides from the TB-specific antigens ESAT-6 or CFP-10. The interferon gamma released by single cells was observed as spots. Automated spot counting was performed using an ELISpot plate reader (AID, Strassberg, Germany). At the time of performing and reading the assays, persons responsible for reading the test were blind to TST results and other health related information.

#### Tuberculin skin tests

TST is unlikely to induce false positive T-SPOT.TB responses [26], but might enhance sub-threshold responses, so it was performed after drawing blood for the repeat T-SPOT.TB assay at the follow-up study visit, three weeks after the five-year visit. As recommended by the manufacturer, 2 tuberculin units of RT-23 purified protein derivative (PPD) (Statens Serum Institut, Copenhagen, Denmark) were injected intradermally into the forearm and the diameter of induration was read 48–72 hours later. Tuberculin injections were performed along the longitudinal axis of the forearm, and the diameter of reaction was measured transversely. Reaction sizes greater or equal to 10 mm were considered positive, based on evidence from studies in Malawi that reactions of this size are associated with increased risk of tuberculosis disease in both BCG-immunised and non-immunised individuals, implying that these individuals are latently infected with *M.tb* [27]. The team performing the TSTs was trained by a highly experienced nurse. Dual readings of a proportion of the TSTs (27.9%) were made by two trained study nurses. The readings were made independently by the two nurses and were not shown subsequently to either reader. They showed a very close agreement between the readers: there was a difference in induration in only two of the readings and in both cases the difference was 1 mm. Because of this close agreement, only the readings which were made by the first reader will be included in our analysis. Tuberculin was kept under refrigeration when not in use.

### Data Analysis

Personal data and TST results were captured into Microsoft Access databases. Data were double-entered and then checked by the study data manager for integrity and consistency. Spot counts were retrieved from the automated plate reader and entered into Microsoft Excel. In accordance with the manufacturer’s instructions, T-SPOT.TB responses were considered positive if either or both of panel A (containing ESAT-6 peptides) or panel B (containing CFP-10 peptides) had six or more spot forming units (SFUs) above the negative control when the negative control had five or less SFUs. In cases where the negative control had six to 10 SFUs, the result was defined as positive when either the ESAT-6- or CFP-10- stimulated well contained at least twice as many SFUs as the negative control well. The result was considered indeterminate if the positive control had less than 20 SFUs (unless either panel A or panel B was positive, as described above, in which case the result was valid), or if the negative control well had 10 or more SFUs. Data analysis was performed with STATA 10.0 (StataCorp, College Station, Texas, USA). Concordance between baseline and repeat T-SPOT.TB, and between T-SPOT.TB and TST was calculated using the kappa (κ) statistic, and assessed according to the criteria suggested by Landis and Koch [28]. Strength of association between SFUs recorded for the two RD1 antigens was estimated by calculating Spearman’s rank correlation coefficient (r_s_). Proportions with positive TST results were compared between groups defined by T-SPOT.TB results using a chi-squared test. The chi-squared test was also used to compare the number of participants for whom the two T-SPOT.TB results agreed, and for whom T-SPOT.TB and TST results agreed, between household contacts and non-household contacts. A 5% significance level (two-sided p = 0.05) was used for all tests.

## Results

### Characteristics of the Study Population

**Figure 1:**
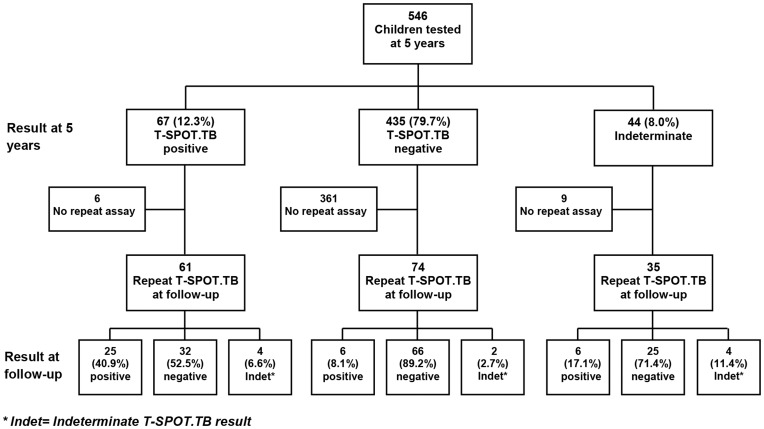
T-SPOT.TB assay results at five years and at follow-up: Protocol 1.

**Figure 2:**
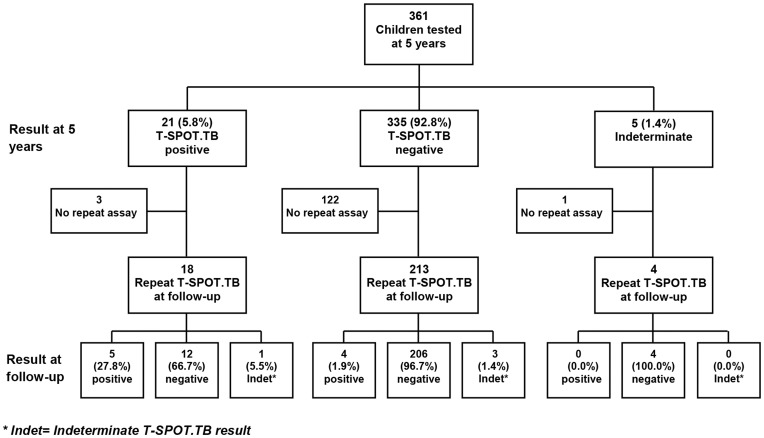
T-SPOT.TB assay results at five years and at follow-up: Protocol 2.

The Entebbe Mother and Baby Study (EMaBS) recruited 2507 pregnant women. Information was obtained on 2345 live births and 1622 were under follow-up at five years, of whom 1438 were seen by the study physicians at age five. Of these, 907 took part in the T-SPOT.TB study. Of the 531 who did not take part in the study, 186 had passed age five before the study began, the parents or guardians of 237 did not give consent, and 108 had moved outside the study area and could not comply with the required procedures. Mothers of participants who took part in the T-SPOT.TB study were on average older, more educated, and of higher socio-economic status than mothers of those who were not included in this study. The participants themselves were less likely to be HIV infected than those who were not included in this study. Of the 907 participants, 432 (47.6%) were female and 475 (52.4%) were male. In total, 56 (6.2%) were household contacts of TB patients, defined as being reported to have been in household contact with a known TB case at any time during the past five years. Analysis of anthropometric data showed a low level of undernutrition, with prevalence of wasting (weight-for-height z-score <−2) at 5.4%. Only 13 (1.4%) of the participants were HIV infected, and prevalence of other infections was relatively low, with 4.0% of children having asymptomatic malaria infection and 9.6% of children having a helminth infection. Where relevant, we assessed the results from Protocol 1 (before amendment; 546 participants), and Protocol 2 (after amendment; 361 participants) separately. The flow of participants in Protocol 1 and Protocol 2 is shown in Figure 1 and Figure 2. The prevalence of wasting was higher for children recruited under Protocol 1 compared to those recruited under Protocol 2 (prevalence 7.6% versus 1.9%, p = 0.001). The prevalence of HIV, asymptomatic malaria, and any helminth infection did not differ between the two Protocols (p = 0.92, 0.46 and 0.49, respectively). Only one child, among those that were household TB contacts, or those that were T-SPOT.TB and/or TST positive, was diagnosed with active TB.

### T-SPOT.TB at Five Years and at Follow-up

Overall, at five years, 88 (9.7%) children were positive for T-SPOT.TB, 770 (84.9%) were negative, and the remaining 49 (5.4%) were indeterminate. We re-examined 405 children at follow-up approximately three weeks later (Figure 1 and Figure 2). Excluding indeterminate results from both tests, 356 children were eligible for direct comparison between the baseline and the repeat T-SPOT.TB assay. For both protocols, agreement between T-SPOT.TB at five years and T-SPOT.TB at follow-up was weak (protocol 1, κ = 0.37; protocol 2, κ = 0.35). More than half of those that were positive on the first test were negative on the second in both protocols, whereas, excluding indeterminate results, 96% of the baseline negatives were consistently negative at follow-up (Figure 1 and Figure 2).

In order to ascertain whether spot counts were concentrated around the diagnostic test cut-off level for participants whose T-SPOT.TB result varied between age five and follow-up, we analysed median changes in spot counts for each RD-1 antigen according to whether participants were T-SPOT.TB positive on the first test and negative on the second test (+/−) or T-SPOT.TB negative on the first test and positive on the second (−/+) (Table 1). Although some spot counts were close to the cut-off on the repeat assay, many of the children who were either +/− or −/+ had SFUs considerably above the cut-off for their positive test: the median change in spot counts between the two tests ranged from six to 17.5 SFUs.

**Table 1:** Median difference in spot counts to ESAT-6 and CFP-10 for participants whose T-SPOT.TB result varied between age five and follow-up.

	T-SPOT.TB result	Observations	Median difference in SFUs	IQR
	Initial/follow-up			
**ESAT-6**				
Protocol 1	+/−	32	−12.00	−32.25, −4.00
	−/+	6	10.50	3.75, 13.50
Protocol 2	+/−	12	−9.50	−12.00, −7.25
	−/+	4	6.50	1.25, 11.00
**CFP-10**				
Protocol 1	+/−	32	−17.50	−25.75, −8.50
	−/+	6	6.00	2.5, 17.75
Protocol 2	+/−	12	−6.50	−11.75, −3.00
	−/+	4	7.00	2.25, 11.00

SFUs: spot forming units; IQR: interquartile range; + indicates positive result; – indicates negative result.

### T-SPOT.TB and TST at Follow-up

Having demonstrated that variations between the initial and repeat T-SPOT.TB results were not characteristic only of protocol 1, we combined results from both protocols to compare T-SPOT.TB and TST results. Data for this analysis were therefore biased towards children with a positive first T-SPOT.TB result, and with a history of contact with a TB patient. Results from all three tests (T-SPOT.TB at five years and at follow-up, and TST at follow-up) were available for 319 children (Table 2). Agreement between T-SPOT.TB and TST was weak (κ = 0.28 for T-SPOT.TB assay at 5 years and κ = 0.40 for T-SPOT.TB at follow-up). Combining T-SPOT.TB results, among children who were negative on both T-SPOT.TB tests, 5 (2.2%) had a positive TST; of those who were positive on only one T-SPOT.TB test, 4 (8.2%) had a positive TST; and of those who were positive on both tests, 14 (50%) had a positive TST (p<0.001: Table 2). The 14 children who were positive on all three tests constituted only 4.4% of the 319 children who had all three tests done.

**Table 2:** Agreement between T-SPOT.TB and TST results.

		TST result		
T-SPOT.TB result at 5 years	T-SPOT.TB resultat follow-up	–	+	Total
–	–	218 (97.8%)	5 (2.2%)	223
–	**+**	8 (88.9%)	1 (11.1%)	9
**+**	**–**	37 (92.5%)	3 (7.5%)	40
**+**	**+**	14 (50.0%)	14 (50.0%)	28
Indeterminate at either time point		18 (94.7%)	1 (5.3%)	19

+ indicates positive result; – indicates negative result.

We evaluated sizes of TST induration among children who had differing T-SPOT.TB and TST results (Table 3), to establish whether discordant results were associated with sizes close to the 10 mm cut-off. Children who were TST positive but T-SPOT.TB negative at both time points tended to have TST indurations close to the cut-off. By contrast, all but one of the 14 children who were TST negative but T-SPOT.TB positive at both time points had no detectable induration. Children who were T-SPOT.TB positive at only one time point had large sizes of TST induration if they were TST positive, but small or no detectable induration if they were TST negative. These results paint a general picture that the high level of disagreement between T-SPOT.TB and TST was not due to threshold TST values.

**Table 3:** Assessment of size of TST in relation to T-SPOT.TB and TST results.

T-SPOT.TB resultat 5 years	T-SPOT.TB result at follow-up	TST result	Number of children	Median size of TST induration (mm)	IQR
**–**	**–**	**–**	218	0.0	0.0, 0.0
**–**	**–**	**+**	5	11.0	10.0, 13.0
**+**	**+**	**+**	14	15.0	13.7, 17.5
**+**	**+**	**–**	14	0.0	0.0, 0.0
positive at either time point		**+**	4	16.5	11.0, 19.7
positive at either time point		**–**	45	0.0	0.0, 0.0

IQR: interquartile range; + indicates positive result; – indicates negative result.

### Correlation between RD1 Antigens

**Figure 3:**
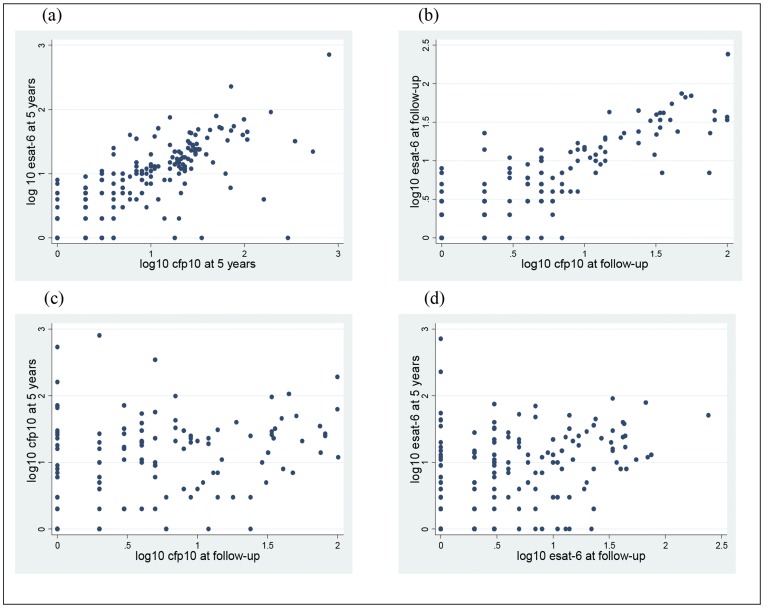
Association between RD1 antigens at five years and at follow-up. Results are presented as logs (to base 10) of spot forming units for the two RD1 antigens. (a) ESAT-6 at five years vs.CFP-10 at five years (n = 179); r_s_ = 0.7548 (b) ESAT-6 follow-up vs. CFP-10 follow-up (n = 158); r_s_ = 0.7838 (c) CFP-10 at five years vs. CFP-10 at follow-up (n = 127); r_s_ = 0.3125 (d) ESAT -6 at five years vs. ESAT-6 at follow-up (n = 163); r_s_ = 0.3576.

To determine whether there was a correlation between ESAT-6 and CFP-10 RD1 antigens, we analysed spot counts in greater detail (Figure 3). Correlation between ESAT-6 and CFP-10 spot counts at five years was high (r_s_ = 0.75), as was correlation at follow-up (r_s_ = 0.78). By contrast, correlation between ESAT-6 spot counts at five years and at follow-up was low (r_s_ = 0.35), as was correlation between CFP-10 spot counts at five years and at follow-up (r_s_ = 0.31). Thus, ESAT-6 and CFP-10 strongly correlated with each other at a given time point, but each individual antigen correlated poorly with itself at the two distinct time points. This was also observed when analysis of spot counts was done separately for each protocol (data not shown). These results indicate that the same individuals were responding to both RD1 antigens at a given time point, but that different individuals were responding at different time points. This was consistent with the high levels of unstable responses observed between baseline and follow-up.

### Household Contacts of TB Patients

We enrolled 56 household contacts (HHC) of TB patients at five years. Of these, 41 had a repeat T-SPOT.TB test at follow-up and a TST result was available for 35. Excluding indeterminate results, there was better agreement between initial and follow-up T-SPOT.TB results among household TB contacts than among non-contacts (κ = 0.77 and 0.39 respectively; p = 0.15): of those HHC who were positive at five years, 70% were also positive at follow-up, and all the HHC who were negative at five years were also negative at follow-up. However, agreement between positive T-SPOT.TB and TST was still weak: of the six HHC who were positive on both T-SPOT.TB tests, only two had a positive skin test. All the 27 HHC who were negative on both T-SPOT.TB tests were also negative with the TST. Household TB contacts also experienced somewhat higher proportions of T-SPOT.TB positivity (17.8% vs. 9.2%; p = 0.045 at baseline and 17.1% vs. 10.7%; p = 0.265 at follow-up) and TST positivity (11.4% vs. 7.0%; p = 0.353), than non-contacts. Age of first exposure was available for 36 (64%) HHC. Five children were exposed as infants, two as one-year olds, seven as three-year olds, five as four-year olds and 17 were exposed in the year preceding enrolment in this study. There was no evidence of a trend in probability of a positive test result with age of exposure for T-SPOT.TB (p = 0.44 at enrolment, p = 0.36 at follow-up) or for TST (p = 0.80).

### Tuberculosis Infection Prevalence Estimates

The infection prevalence estimates by different measures are shown in Table 4. A single T-SPOT.TB result at five years would estimate infection prevalence at 9.7% among children in our study, whereas a more stringent criterion of a positive TST and positive T-SPOT.TB at both time points would put the prevalence at 1.5%. From our data, the true infection prevalence in this population of children lies somewhere between 1.5% and 11.2%, highlighting the complexity of estimating LTBI in this age group and setting, using T-SPOT.TB and/or TST.

**Table 4:** *Mycobacterium tuberculosis* infection prevalence estimates by different measures.

Measure of infection	Number	Prevalence estimate (95% CI)
Positive on all three tests (T-SPOT.TB at baseline and follow-up, TST at follow-up)*	14	1.5% (0.8%–2.6%)
Positive T-SPOT.TB result at either or both time points, and a positive TST**	18	2.0% (1.2%–3.1%)
Positive T-SPOT.TB result at both time points*	30	3.3% (2.2%–3.7%)
Positive T-SPOT.TB result at baseline	88	9.7% (7.9%–11.8%)
Any one positive T-SPOT.TB result**	98	10.8% (8.9%–13.0%)
Any one positive result (T-SPOT.TB or TST)**	102	11.2% (9.3%–13.5%)

The denominator is 907 for all the estimates.

* All but 9 of the baseline T-SPOT.TB positives were followed up. Prevalence doesn’t change when the 9 are subtracted from the denominator.

** Does not take into account the baseline T-SPOT.TB negatives that were not followed up, yet might have turned positive on follow-up. This is not likely to change the prevalence estimates, since most (96%) of the baseline negatives that were followed up remained negative.

## Discussion

Our aim was to use the T-SPOT.TB assay to determine *M.tb* infection prevalence among *M.tb* exposed and unexposed five year old children in Entebbe, Uganda. We anticipated that positive responses would be confirmed with a second T-SPOT.TB assay and a TST. However, we have demonstrated a high level of instability in positive T-SPOT.TB responses between baseline and a three week follow-up and poor agreement between T-SPOT.TB and TST responses, making the categorisation of children as LTB-infected or LTB-uninfected difficult.

Analysis of SFUs showed that positive responses were not concentrated around the diagnostic test cut-off level for children whose T-SPOT.TB result varied between the first and second tests. Instead, some children showed large changes in response – both increases and decreases in SFUs – between the two assessments. In contrast, ESAT-6 and CFP-10 spot counts correlated well at each time point. Together these findings suggest that our results may reflect a true change in the immune response to the *M.tb* antigens in peripheral blood. Several other studies, albeit with longer term follow-up, have observed variations in responses between baseline and follow-up [14], [21], [29]. In a bid to explain changes on follow-up when using IGRAs, Hill *et al.* suggested that IGRA responses are not long-lived and generally require sustained, continuous exposure to *M.tb* antigens to maintain high frequencies [14]. They hypothesized that the decline in response following exposure to *M.tb* may be a reflection of the lifecycle of *M.tb* and the dynamic interaction with the host immune system. As the mycobacteria enter a state of dormancy, secretion of ESAT-6 and CFP-10 may decline leading to a decrease in circulating memory T cells specific for the antigens used in the assay [14], [30]. The weak association between T-SPOT.TB and TST in our study raises a further question as to whether the responses observed indicate LTBI at all. It may be that the unstable positive T-SPOT.TB responses in this young age group reflect a weak (perhaps transient) response to *M.tb* antigens in BCG-immunised children who resist the establishment of latent infection. However, the short time between the two assessments in our study make it unlikely that a change in TB exposure could account for the effects observed.

Some studies have described a rather high rate of changes from negative to positive IGRA response during follow-up in high-risk populations in endemic settings [29], [30]. Incident infections might explain this, especially in studies involving contacts of TB patients, or, again, the intermittent secretion of ESAT-6 and CFP-10 by *M.tb*. However, in our study, changes from negative to positive were very few compared to changes from positive to negative.

Fluctuation in IGRA results upon serial testing has sometimes been attributed to technical factors. These factors may include blood volume (for QuantiFERON assays), different staff performing the assay, preanalytical delays and reagents. The T-SPOT.TB assay uses blood volumes as low as 2 ml; we collected 4 ml on average. Most of the assays were performed by the study laboratory technologists (GN and JEL), and any other staff performing the assay were given specialist training and worked under the supervision of the study laboratory technologists. Doberne *et al.* [31] recently demonstrated that preanalytical delay resulted in increased positive-to-negative reversions in as little as six hours. Such delays were not characteristic of our study (median time after sample collection = 1.4 hours, IQR 50 min-2.2 hours), so time to processing is unlikely to explain our observed fluctuations. A recent systematic review [20] showed that tuberculin skin testing has a boosting effect on IGRA responses, but this cannot explain the observed fluctuations in our study because we drew blood before performing the TST.

Longitudinal assessments for IGRAs in published literature have mainly been in contacts of TB patients [11], [29], [32], [33]. Our study investigated children, most of whom had no known TB contacts. We hypothesize that the intense exposure that TB contacts experience, compared to non-contacts, may explain the more stable responses observed in those studies. In keeping with this hypothesis, we found that household TB contacts in our study showed better agreement between the baseline and repeat test although the number of contacts was too small to provide good power for sub-group analyses.

T-SPOT.TB has been dubbed the “100-year upgrade” to the well-established TST for the diagnosis of TB infection [34]. However, we report a high level of disagreement between the TST and T-SPOT.TB in our cohort of children. Previous studies have shown that levels of agreement are varied depending on the study and outcome measurement. For example, in four investigations that analysed agreement between the two tests, the κ scores ranged from -0.15 to 0.76 [16], [35]–[37]. In our group, recent unpublished data from adult women in Entebbe showed that T-SPOT.TB performed better as an indicator of LTBI among adults. Among 23 women who tested T-SPOT.TB positive, 21 (91%) were TST positive as well. All these findings support the perception that agreement between the TST and the IGRA in the diagnosis of TB infection might vary depending on several factors such as age, history of previous BCG vaccination [38], and infection with other mycobacteria.

The immunological inferences that can be drawn from the observed discordance between T-SPOT.TB and TST in our study are unclear. TST results may be falsely negative in children due to the influence of factors such as malnutrition, concurrent viral and/or parasitic infections, and concurrent medical conditions and diseases [39]. However, these factors were not characteristic of our participants. For example, we had TST data for six of the 13 HIV positive participants, and none of these had discordant T-SPOT.TB and TST results. The children in this study were BCG-immunised, making it possible that the discordances observed were due to false TST positives, but this is unlikely because we observed fewer positives by TST than by T-SPOT.TB. Furthermore, there was a general lack of intermediate sized TST responses, which have been attributed to BCG vaccination and infection with mycobacteria other than tuberculosis [40]–[42]. The discordances may therefore be due to the unstable T-SPOT.TB responses between baseline and follow-up, rather than a result of the known shortcomings of the TST.

Tuberculin surveys carried out in the 1970s suggested that the annual risk infection (ARI) with TB in Uganda was approximately 3%, although rates were lower than this in young children [43]. Recent national reports and other studies continue to quote this estimate [44]–[47] although some surveys have indicated a steady decline in ARI – for example, a survey in northern Uganda in 1994 estimated ARI at 1.4% [48]. Our results suggest that this may now be a more realistic figure for young children in Central Uganda also. However, our study cohort was derived from a small area, and those who participated in this study showed some biases. New *M.tb* infection surveys of broader scope may be warranted for Uganda.

The principal limitation of our study was the change in protocol, resulting in variation in sample sizes for T-SPOT.TB at five years (n = 907), repeat T-SPOT.TB at follow-up (n = 405) and TST (n = 319) making it difficult to compare the three tests directly. Secondly, the proportions of children followed up were different depending on the child’s T-SPOT.TB result at five years: this was expected with Protocol 1 but not with Protocol 2, where 85.7% of the children who were initially positive were followed up compared to 63.6% of those who were initially negative. Data analysis was therefore biased towards children with a positive first T-SPOT.TB result. We attempted to overcome this limitation by presenting results from Protocol 1 and Protocol 2 separately where it was relevant, and by comparing the participants’ characteristics between the two protocols.

Our data provide a valuable insight into the usefulness of IGRAs in the diagnosis of TB infection among children living in endemic settings. It has been suggested that recommendations on use of IGRAs in children younger than five years and in immunocompromised children should be taken with caution because of a lack of adequate published data on their efficacy in these groups [49], [50]. Our study has contributed to the increasing evidence that IGRAs may not be superior to TST in children in high incidence settings and cannot be used alone to diagnose TB infection in these settings. Diagnosis of TB infection and estimating TB infection prevalence among children in high incidence settings remains a challenge; better diagnostic tests are still needed.
